# Reaction Systems and Synchronous Digital Circuits

**DOI:** 10.3390/molecules24101961

**Published:** 2019-05-21

**Authors:** Zeyi Shang, Sergey Verlan, Ion Petre, Gexiang Zhang

**Affiliations:** 1 School of Electrical Engineering, Southwest Jiaotong University, Chengdu 611756, Sichuan, China; zeyi.shang@lacl.fr; 2 Laboratoire d’Algorithmique, Complexité et Logique, Université Paris Est Créteil, 94010 Créteil, France; verlan@u-pec.fr; 3 Department of Mathematics and Statistics, University of Turku, FI-20014 Turku, Finland; ion.petre@utu.fi; 4 National Institute for Research and Development in Biological Sciences, 060031 Bucharest, Romania

**Keywords:** reaction systems, synchronous digital circuits, field-programming gate arrays

## Abstract

A reaction system is a modeling framework for investigating the functioning of the living cell, focused on capturing cause–effect relationships in biochemical environments. Biochemical processes in this framework are seen to interact with each other by producing the ingredients enabling and/or inhibiting other reactions. They can also be influenced by the environment seen as a systematic driver of the processes through the ingredients brought into the cellular environment. In this paper, the first attempt is made to implement reaction systems in the hardware. We first show a tight relation between reaction systems and synchronous digital circuits, generally used for digital electronics design. We describe the algorithms allowing us to translate one model to the other one, while keeping the same behavior and similar size. We also develop a compiler translating a reaction systems description into hardware circuit description using field-programming gate arrays (FPGA) technology, leading to high performance, hardware-based simulations of reaction systems. This work also opens a novel interesting perspective of analyzing the behavior of biological systems using established industrial tools from electronic circuits design.

## 1. Introduction

Reaction systems have been introduced in [1] as a formalism for describing the functioning of the living cell by following the interactions between biochemical reactions and the cellular environment, see also [2] for a recent survey. These interactions are fundamentally based on two mechanisms, facilitation and inhibition: the products of reactions may be used by other reactions and may inhibit others, while the environment may add additional ingredients in every step of the process. The framework of reaction systems is a model of biocomputations where the configuration of the system is created by the product of all reactions that were enabled in the previous step, plus the additional contribution of the environment. The facilitation-inhibition mechanism gives reaction systems the ability to follow up explicitly on the cause-effect relationships in a biochemical process, helping to answer questions around why a certain property arises [3,4].

The research on reaction systems has flourished in the last few years along two broad directions. On the one hand, reaction systems have been investigated for their mathematical and computational properties as a model for interactive biocomputation, on topics such as minimal systems [5,6], functions and state sequences [7,8], timed versions [9,10,11,12], modular decompositions [13], equivalence properties [14,15,16]. On the other hand, reaction systems have been studied a modeling framework for capturing realistic biological processes and for enhancing their analytic capabilities; topics include model checking properties [17,18,19], modeling of the heat shock response [20], of the self-assembly of intermediate filaments [21], and of the ErbB signaling pathway [22], bifurcation and multi-stability properties [23].

There has been a growing interest also in the simulation of the behavior of reaction systems, supporting both lines of research described above. There are currently two different simulators of interactive processes of reaction systems. The brsim/WEBRSIM simulator is a Haskell-based implementation with a user-friendly web interface, providing also a few simple model checking analysis options. It is currently the fastest available central processing unit (CPU)-based simulator. The HERESY simulator [22] is based on a graphics processing unit (GPU) implementation with compute unified device architecture (CUDA) and is especially efficient for very large reaction systems with hundreds of reactions. It also has a slower CPU-based implementation.

The main contribution of this paper is the first attempt to implement reaction systems in the hardware. We first present the links between two different models: reaction systems and synchronous digital circuits. Our main observation is that interactive processes in reaction systems are similar with the calculations of digital circuits and we establish this in a formal sense. Based on this we build a new hardware-based simulator for reaction systems by simply translating a reaction system into a digital synchronous circuit, and simulate it using a fast FPGA-based implementation. This implementation is much faster than the previous software-based simulators, bringing speeds of more than $10^{8}$ steps per second, thus allowing a speed-up of order $2.5\times 10^{5}$ with respect to best existing hardware (and software) implementations. Hence, this work shows us how to perform high-speed and large-scale simulations of reaction systems, which opens a way for an efficient investigation of big biological models, like for example the ErbB signaling pathway [24]. This can potentially speed-up the drug discovery by pointing out different possible drug targets having the maximal desired effect helping to target the further experiments [25,26]. At the same time, this paper is the first study that shows how formal tools like Mealy and Moore automata can be used to investigate the behavior of reaction systems and thus of corresponding biological systems. This also allows to use high-quality industrial tools from the circuit design area to analyze and optimize the obtained circuits (thus the initial system). By performing slight modifications, the proposed method can be used to simulate arbitrary Boolean networks, which opens many interesting perspectives. While there are several works on FPGA simulation of Boolean networks, e.g., [27,28,29], our article differs from them by highlighting the theoretical link between the model of Boolean networks and sequential switching circuits, hence implementing the corresponding simulation in most efficient way.

## 2. Preliminaries

We assume basic familiarity with the notions of Boolean (switching) functions and formulas. There are many books available presenting these notions, we suggest [30] for an introduction. Below we recall the differences between Boolean and switching algebras and circuits.

A Boolean algebra [31] is a distributive and complemented lattice. A switching algebra firstly developed by Shannon in [32] can be seen as a restriction of a Boolean algebra to two elements: 0 and 1. It is also called a two-element Boolean algebra. The primary applications of switching algebra are in digital circuit design and Boolean (two-valued) logic, see [33] for more details. Traditionally, in switching algebra symbols ·, +, and ′ are used for conjunction, disjunction and negation, respectively. In this paper we will use the standard logical notations for these functions: ∨, ∧, and $\bar{x}$.

A switching function is any expression in switching algebra. The evaluation of the function is done in an ordinary way by assigning values 0 or 1 to corresponding arguments and after that evaluating the result by performing ordinary Boolean transformations. A switching circuit having *n* inputs and *m* outputs is computing a switching function of the form $f:{\left\{0,1\right\}}^{n}\to {\left\{0,1\right\}}^{m}$.

We remark that in the present-day literature the term switching is often replaced by Boolean, which has the meaning of the former one.

### 2.1. Sequential Circuits

There are two types of (switching) circuits: combinatorial, where the value of the output is a function of only current input values and sequential, where the value of the output depends on the input and also on the state of the circuit. The state allows us to memorize past values and to perform decisions based on the partial history of the computation. Hence, in this case the value of the function may be different for same inputs at different time steps (usually corresponding to the master clock pulses that drive the circuit).

So the functioning of a sequential switching circuit with *n* inputs and *m* outputs and *s* binary-state variables can be described by the following equations:

(1) $$\begin{array}{cc} Q(t+1) & =F(Q(t),X(t))\\ Y(t) & =G(Q(t),X(t)), \end{array}$$

where $X\left(t\right)=(x_{1}\left(t\right),\cdots ,x_{n}\left(t\right))$ is the vector of input variables at time t≥0, $Y\left(t\right)=(y_{1}\left(t\right),\cdots ,y_{m}\left(t\right))$ is the vector of output variables at time *t*, $Q\left(t\right)=(q_{1}\left(t\right),\cdots ,q_{s}\left(t\right))$ is the vector of internal states at time *t*, $F:{\left\{0,1\right\}}^{s}\times {\left\{0,1\right\}}^{n}\to {\left\{0,1\right\}}^{s}$ and $G:{\left\{0,1\right\}}^{s}\times {\left\{0,1\right\}}^{n}\to {\left\{0,1\right\}}^{m}$.

Each sequential circuit is associated with a truth-table with its columns headed (in order):

Q(t);X(t);Q(t+1);Y(t).

An equivalent description of such circuits was shown by Mealy using Mealy automata [34]. It corresponds to a finite state automaton with input and output where the transitions are labeled by two values corresponding to the input and the output. The transition is applicable if the current input corresponds to the one indicated on the transition, and then the automaton outputs the corresponding output value. A similar representation can be done by Moore automata [35]. In this case the output depends only on the previous state and it is indicated beside the state label. Both models are equivalent, however there is a one step output delay if using Moore model.

**Example** **1.** *Let us consider the example presented in [34]. The sequential circuit is described by the following equations (n=1, m=1 and s=2).*

(2) $$\begin{array}{cc} q_{1}\left(t+1\right) & ={\bar{q}}_{1}\left(t\right)\wedge {\bar{q}}_{2}\left(t\right)\vee {\bar{x}}_{1}\left(t\right)\wedge {\bar{q}}_{2}\left(t\right)\\ q_{2}\left(t+1\right) & =q_{1}\left(t\right)\wedge {\bar{q}}_{2}\left(t\right)\vee x_{1}\left(t\right)\wedge q_{1}\left(t\right)\\ y_{1}\left(t\right) & ={\bar{q}}_{1}\left(t\right)\wedge {\bar{q}}_{2}\left(t\right). \end{array}$$

**Figure 1:**
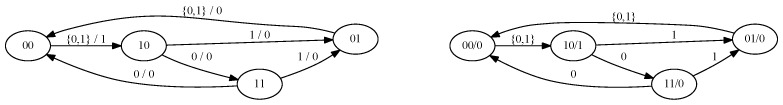
The Mealy (**left**) and Moore (**right**) automata for the circuit described by Equation (2). The label of the state corresponds to the value of the vector $\left(q_{1},q_{2}\right)$. In the case of Moore automaton it additionally contains the value of the output variable *y*. The label of the transition corresponds to the value of the input variable *x*. In the case of Mealy automaton, it additionally contains the value of the output variable *y*.

*These equations correspond to the truth table (computed by listing all possible combinations of values for $q_{1}\left(t\right)$, $q_{2}\left(t\right)$ and x(t)) presented in Table 1. The corresponding Mealy and Moore automata are depicted on Figure 1. It can be seen that these automata are a convenient way to represent the truth table. For example, being in state* 10 *and following a transition labeled by* 0, *yields the state* 11 *and the output* 0 *(at the next step in the case of Moore automaton), corresponding to the fifth line of the truth table.*

### 2.2. Reaction Systems

Below we briefly recall the definition of reaction systems given in [1,2].

**Definition** **1.** *A reaction is a triplet a=(R,I,P), where R,I,P are finite nonempty sets with R∩I=∅. If S is a set such that R,I,P⊆S, then a is a reaction in S.*

Informally, reaction allows us to define causal effects between the production of the result *P* and the presence of reactants *R* and the absence of inhibitors *I*. Formally, we defined $res_{a}\left(X\right)=P$, if and only if R⊆X and I∩X=∅. This operation can be generalized to a set of reactions A in a standard manner. Finally, the set of all reactions over a set *S* is denoted as rac(S).

**Definition** **2.** *A reaction system (RS), is an ordered pair A=(S,A) such that S is a finite set, and A⊆rac(S).*

**Definition** **3.** *Let A=(S,A) be a RS and let n≥0 be an integer. An (n-step) interactive process in A is a pair π=(γ,δ) of finite sequences such that $\gamma =C_{0},\cdots ,C_{n}$ and $\delta =D_{0},\cdots ,D_{n}$, where $C_{0},\cdots ,C_{n},D_{0},\cdots ,D_{n}\subseteq S$, $D_{0}=\emptyset$, and $D_{i}=res_{A}\left(D_{i-1}\cup C_{i-1}\right)$ for all i∈{1,⋯,n}.*

Informally, an interactive process allows to compute a time series of the values of variables from *S* based on the input provided at each step *t* by the context $C_{t}$.

The reaction systems model abstracts away from the various numerical details of biochemical reactions, and rather it only indicates whether a resource is present or not in the system, and how they trigger or block the execution of a certain reaction. This is best described as seen in the definitions above through a set-theoretical framework, where each configuration of the system is a subset of the reactant set. The environment is an active present of the semantic of reaction system and in each step of the interactive process of a reaction system, it contributes potentially new, additional resources to the current configuration. The current configuration then determines the next one by triggering all the enabled reactions and having them generate through their products the next configuration of the system. All the currently existing resources are excluded from the next configuration, unless they were produced by one of the enabled reactions. This is the basic “non-permanency” principle of reaction systems, proposing the idea that maintaining a resource in a system is a matter that has to be actively supported by the system.

Many versions of reaction systems have been proposed, adding various features to the basic model, such as a numerical dimension of the reactants, describing how many are available in a configuration [9], and systems with durations, where there is an explicit, potentially non-zero life duration of a reactant, keeping it into the system for several steps without additional support from the enabled reactions [10]. All of these versions were proved to be equivalent with the basic model from the point of view of their computing power [2]. We focus in this article only on the basic version of the reaction systems, as defined above.

## 3. Results

In this section we will show how it is possible to transform a reaction system to a switching circuit and conversely.

### 3.1. From Reaction Systems to Switching Circuits

First we introduced a normal form for a reaction system under an interactive process.

**Definition** **4.** *A reaction system A=(S,A) is said to be in a normal form with respect to the interactive process π=(γ,δ) if*

- *for any a=(R,I,P)∈A it holds |P|=1 (i.e., only one product is allowed per reaction),*
- *$\underset{(R,I,P)\in A}{⋃}P\cap \underset{i\geq 0}{⋃}C_{i}=\emptyset$ (i.e., the set of products is disjoint with the set of contexts).*

**Theorem** **1.** *For any reaction system A=(S,A) and any interactive process π=(γ,δ) such that C⊆Z, for any C∈δ it is possible to construct an iterative process $\pi^{\prime }=\left(\gamma ,\delta^{\prime }\right)$ and a reaction system $A^{\prime }=\left(S^{\prime },A^{\prime }\right)$ in normal form with respect to $\pi^{\prime }$ such that δ is a projection of $\delta^{\prime }$ with respect to S.*

**Proof.**  Initially $S^{\prime }=S$. First we add to $A^{\prime }$ all reactions from *A* that have a single product.

Next, if a=(R,I,P) with |P|>1, than we add to $A^{\prime }$ the reactions (R,I,x),x∈P.

Let $T=\{x|\left(R,I,x\right)\in A^{\prime }\}$. Now, if there is a symbol X∈Z∩T then we add a new background symbol $X^{\prime }$ to $S^{\prime }$ and we replace the reaction (R,I,X) by $\left(R,I,X^{\prime }\right)$. We also add to $A^{\prime }$ the set of reactions $\left\{\left(R\backslash \left\{X\right\}\cup \left\{X^{\prime }\right\},I,P\right)|X\in R\right\}\cup \left\{\left(R,I\backslash \left\{X\right\}\cup \left\{X^{\prime }\right\},P\right)|X\in I\right\}$ (i.e., we made a copy of each reaction involving *X* replacing it by $X^{\prime }$).

Now consider the interactive process $\pi^{\prime }$, which is obtained by feeding $A^{\prime }$ the contexts γ. Obviously, the reaction system $A^{\prime }$ is in the normal form with respect to $\pi^{\prime }$. Moreover, because no rule was deleted and the added rules introduce primed symbols, which act as aliases for their non-primed versions it is easy to see that the projection of $\delta^{\prime }$ over *S* yields δ.  □

We can define the input for a reaction system inp(A) as the set of all possible context symbols: inp(A)={Z∈δ∣foranyinteractiveprocessπ={γ,δ}}. Similarly, we might be interested by particular symbols that can appear in the result. So we define can define the output of a RS as a projection of *S*: out(A)⊆S.

Now we construct the Equation (1) using the method from [1], that transforms a reaction system to a Boolean formula in disjunctive normal form (DNF).

Suppose that we have a reaction system A=(S,A) in a normal form with input I. Then each group of reactions having the same product $A_{p}=\left\{\left(R_{a},I_{a},p\right)|a\in A\right\}$ can be seen as the following equation:

(3) $$p\left(t+1\right)=\underset{\left(R_{a},I_{a},p\right)\in A_{p}}{⋁}\left(\underset{X\in R_{a}}{⋀}X\left(t\right)\wedge \underset{Y\in I_{a}}{⋀}\bar{Y}\left(t\right)\right).$$

In order to compute the output of the switching circuit we add the following equation (since it is a projection, we may omit it if it is clear from the context):

(4) y(t)=y(t),forally∈out(A).

Equations (3) and (4) are of the form of Equation (1), so they define a switching circuit.

Moreover, since sets of reactants and inhibitors are disjoint, formula (3) is in DNF.

**Example** **2.** *Consider the reaction system $A_{n}=\left(S_{n},A_{n}\right)$, n>1 from [1] that defines a binary counter. For n>1, let $S_{n}=\left\{e_{0},\cdots ,e_{n}\right\}$. The set of reactions is defined as follows.*

$$\begin{array}{ccc} A_{n} & = & \left\{(\left\{e_{i}\right\},\left\{e_{j}\right\},\left\{e_{i}\right\})|0\leq j<i\leq n\right\}\cup \\ & & \left\{(\left\{e_{0},\cdots ,e_{i-1}\right\},\left\{e_{i}\right\},\left\{e_{i}\right\})|0<i\leq n\right\}. \end{array}$$

*In the interactive process $e_{0}$ is the input and $e_{i}$, 1≤i≤n are the output symbols. So the system is in the normal form. We construct the equations for the switching circuit:*

$$\begin{array}{c} e_{i}\left(t+1\right)=\underset{0\leq j<i\leq n}{⋁}\left(e_{i}\left(t\right)\wedge {\bar{e}}_{j}\left(t\right)\right)\vee \underset{1\leq i\leq n}{⋁}\left({\bar{e}}_{i}\left(t\right)\wedge \underset{0\leq k\leq i-1}{⋀}e_{k}\left(t\right)\right) \end{array}$$

**Figure 2:**
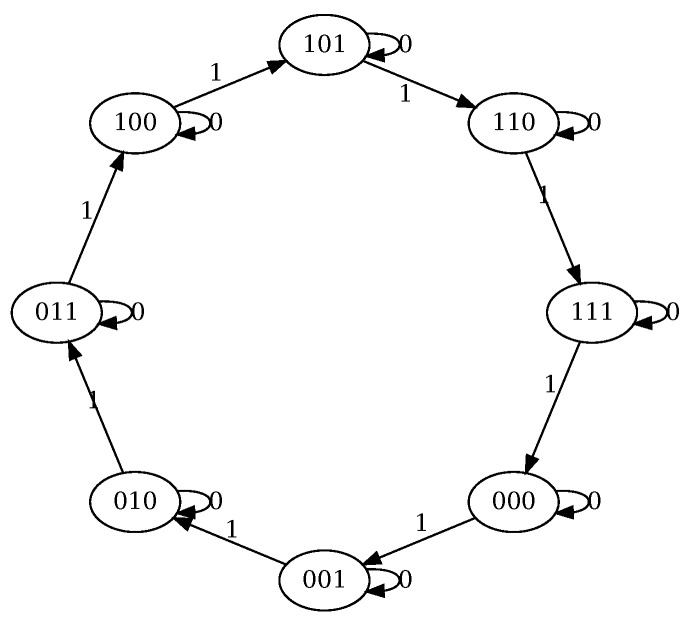
Moore machine for Example 2 and n=3. The state label corresponds to the values of the vector $\left(e_{3},e_{2},e_{1}\right)$ and the transitions are labeled by the value of $e_{0}$. The output is the label of the state.

*The corresponding Moore machine for n=3 is given on Figure 2. From the picture it can be clearly seen that this circuit implements a binary counter.*

**Example** **3.** *In [21], a model for the self-assembly of intermediate filaments from vimentin tetramers is presented. We consider the first model from that paper (the other more complex variants of the model can be consulted as examples provided with our compiler in [36]). It is defined as follows.*

*The background set is S={O,H,F,d} and the input set is {T}. The reactions are the following (d is the dummy inhibitor):*

$$\begin{array}{c} (\{T\},\{d\},\{O\}),\\ (\{O\},\{d\},\{H\}),\\ (\{H\},\{d\},\{F\}),\\ (\{F\},\{d\},\{F\}). \end{array}$$

*Using Equations (3) and (4) we obtain the following sequential circuit:*

$$\begin{array}{cc} d(t+1) & =0\\ O(t+1) & =T\left(t\right)\wedge \bar{d}\left(t\right)\\ H(t+1) & =O\left(t\right)\wedge \bar{d}\left(t\right)\\ F(t+1) & =\left(H\left(t\right)\wedge \bar{d}\left(t\right)\right)\vee \left(F\left(t\right)\wedge \bar{d}\left(t\right)\right). \end{array}$$

**Figure 3:**
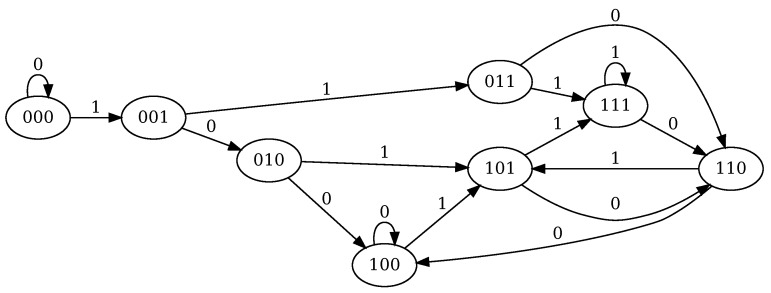
Moore machine for Example 3. The state label corresponds to the values of the vector (F,H,O) and the transitions are labeled by the value of *T*. The output is the label of the state.

*The Moore machine for this circuit is depicted on Figure 3. It can be immediately deduced that there is a steady loop between states* 101, 110 *and* 111 *corresponding to the last rule that keeps F indefinitely once produced.*

### 3.2. From Switching Circuits to Reaction Systems

Now we will show how to construct a reaction system starting from a sequential switching circuit. Let *C* be a circuit with *n* inputs, *m* outputs and *s* internal states described by Equation (1). Without loss of generality we can suppose that *F* and *G* are in disjunctive normal form.

Then we construct a reaction system with input A=(S,A,I), where S=Q∪Y and I=X. The reactions from *A* are defined as follows:

Let *a* be a conjunction $a=a_{1}\wedge \cdots \wedge a_{k}$, k>0. We define

$pos\left(a\right)=\left\{a_{s}|1\leq s\leq k\;\mathrm{and}\;k\;\mathrm{is}\;a\;\mathrm{positive}\;\mathrm{literal}\right\}$

$neg\left(a\right)=\left\{a_{s}|1\leq s\leq k\;\mathrm{and}\;k\;\mathrm{is}\;a\;\mathrm{negative}\;\mathrm{literal}\right\}.$

Then an equation $q_{i}\left(t+1\right)=F_{i}\left(Q\left(t\right),X\left(t\right)\right)={⋁}_{1\leq s\leq p}c_{s}$, where $c_{s}$ is a conjunction of literals from Q(t) and X(t) and p>0 can be written as following set of reactions:

$$(pos\left(c_{s}\right),neg\left(c_{s}\right),\left\{q_{i}\right\}),\;1\leq s\leq p.$$

Now, the initial values of state variables of the circuit give the value $C_{0}$ of the initial context for any interactive process π for this RS. It can be immediately seen that for any sequence of input values for *C*, feeding same values as contexts for A give the same output sequence.

**Figure 4:**
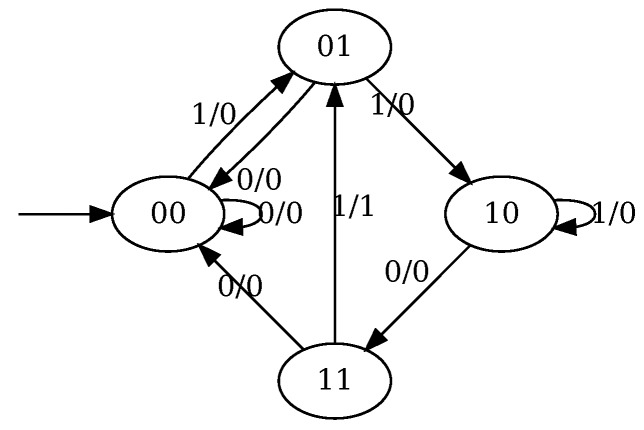
Mealy machine for the 1101 sequence detector. It outputs 1 when the corresponding sequence is encountered as input. The state label corresponds to the vector $\left(q_{2},q_{1}\right)$.

**Example** **4.** *Consider the circuit that implements a sequence detector and outputs 1 if the sequence* 1101 *is detected as input. The corresponding Mealy machine is depicted in Figure 4 and the corresponding truth table is given in Table 2.*

*From this table we can deduce the state equations of the circuit (x being the input bit and y the output result):*

$$\begin{array}{cc} q_{2}\left(t+1\right) & ={\bar{q}}_{2}\left(t\right)\wedge q_{1}\left(t\right)\wedge x\vee q_{2}\left(t\right)\wedge {\bar{q}}_{1}\left(t\right)\\ q_{1}\left(t+1\right) & ={\bar{q}}_{2}\left(t\right)\wedge {\bar{q}}_{1}\left(t\right)\wedge x\vee q_{2}\left(t\right)\wedge {\bar{q}}_{1}\left(t\right)\wedge \bar{x}\vee q_{2}\left(t\right)\wedge q_{1}\left(t\right)\wedge x\left(t\right)\\ y(t) & =q_{2}\left(t\right)\wedge q_{1}\left(t\right)\wedge x\left(t\right) \end{array}$$

*Using the above algorithm these equations are transformed to the following reaction system (where d is the dummy inhibitor and initially the system is empty):*

$$\begin{array}{c} \left\{x\right\}\left\{q_{1},q_{2}\right\}\left\{q_{1}\right\},\\ \left\{q_{2}\right\}\left\{q_{1},x\right\}\left\{q_{1}\right\},\\ \left\{q_{1},q_{2},x\right\}\left\{d\right\}\left\{q_{1}\right\},\\ \left\{q_{1},x\right\}\left\{q_{2}\right\}\left\{q_{2}\right\},\\ \left\{q_{2}\right\}\left\{q_{1}\right\}\left\{q_{2}\right\},\\ \left\{q_{1},q_{2},x\right\}\left\{d\right\}\left\{y\right\}. \end{array}$$

## 4. Discussion

The translation of reaction systems to synchronous circuits gives several advantages. First, the Mealy/Moore machine representation might allow a better understanding of the functioning of the system and of its invariants. Next, there exist many powerful industrial tools allowing the analysis, the minimization and the verification of digital circuits, hence they can be used to transform or minimize the circuit. For example, applying tools from Vivado Design Suite 2018.2 [37] on the translation of the reaction system describing ErbB signaling pathway taken from [22] allowed to reduce the size of the model by 50% by performing cell identification and constant propagation. Finally, the translation to circuits allows us to implement reaction systems in digital hardware in order to perform fast experiments of systems of huge size. In most of the cases, the running speed of FPGA clock can be achieved, yielding a simulation performing at 1–10 ns per step.

The converse translation is also interesting as it allows to use industrial tools for circuit design in order to produce reaction systems. Moreover, many of such tools come with a library of predefined circuits and it should be relatively simple to design complex reaction systems, e.g., simulating a RAM or an Ethernet controller.

Another interesting implication of our research is that, due to the similarity between RS and Boolean networks, the developed method can be directly applied to transform Boolean networks to hardware circuits allowing to use high quality analysis tools and high-speed hardware simulation. This approach is highly scalable, so it can open new perspectives in the area of Boolean biological modeling, providing a way to handle models several orders bigger than those existing in the present.

Finally, the techniques discussed in this work are currently being generalized in order to allow efficient hardware implementations of membrane computing models [38], such as cell-like P systems [39,40,41], bacterial P systems [42] and spiking neural P systems [43,44,45,46,47,48,49], used for processing images [50], controlling mobile robots [51], robot path-planning [52,53], image processing [54] and modeling complex systems [55].

## 5. Materials and Methods

This section discusses the hardware implementation. In order to perform the automated translation of reaction systems to synchronous circuits a compiler RStoVerilog was written that allows to generate hardware description of the circuit using Verilog language. The syntax of the input file for RStoVerilog is the same as for brsim/WEBRSIM reaction systems simulator [56,57] and the description of the command-line options can be found in [36]. As a result it produces a Verilog description of the circuit, a Verilog test bench testing the circuit with the supplied input and the graph describing the corresponding Moore machine in GraphViz format [58]. The program can be found at [36] and can be freely used.

The compiler performed the following steps:

1. Parse the input file.
2. Identify input and output symbols.
3. Duplicate input symbols that are at the same time output symbols.
4. Transform the reaction system into a Boolean circuit in DNF.
5. Write Verilog output (module and the test bench).
6. Optionally, construct Moore automaton of the obtained system.

Step 1 was performed using standard compiling techniques. Now in order to apply the algorithm described in Section 3 one needs to identify input and output symbols. Since the initial concept of reaction systems does not possess this information, we used the following algorithm to automatically identify them. So during Step 2, the program identified symbols that never appeared in the products of a reaction as input symbols, while the others were identified as output symbols. This behavior can be overridden by special annotations in the source file that give the explicit list of input and output symbols (the remaining ones are considered as internal states). Since the above algorithm may lead to symbols that are at the same time input and output symbols, we introduced an additional Step 3, that, for each of these symbols (*x*), created a new input symbol $x_{in}$ and copied all rules involving *x* in the reactants or inhibitors lists replacing *x* by $x_{in}$ for all possible combinations of occurrences. We remark that this step can lead to an exponential blow-up of the number of reactions, so it is strongly suggested to manually identify input and output symbols.

Then Step 4 was performed according to the algorithm described in Section 3. Step 5 is straightforward, as a sequential Boolean (switching) function/circuit in DNF can be directly translated to Verilog. As a result, two files were obtained: a synthesizable (in FPGA) Verilog module containing the code that simulates each step of the reaction system and a (non-synthesizable) test bench that contains the timed update of the input symbols using the provided context.

Finally, the compiler can optionally construct the Moore automaton of the obtained circuit (if “-g” command-line switch is provided). This automaton is iteratively constructed by varying all the inputs starting from the initial state given by the first context. By adding the command-line switch “-ga” all possible initial states are constructed. The result was yielded using the GraphViz format [58]. Due to the combinatorial explosion, it was necessary to construct the automaton (the corresponding algorithm used standard enumeration techniques—so it was exponential), graph generation was limited to systems having less than 32 species. Another reason for this restriction was that bigger graphs were very difficult to analyze visually.

The Verilog test bench obtained by RStoVerilog can be directly simulated in software using a Verilog compiler and simulator. We used Icarus Verilog 10.1 compiler and simulator [59] and the corresponding result is quite competitive with respect to the other existing simulators. In order to make a real-life use case test, we used the reaction system translation of the model of ErbB receptor signal transduction in human mammary epithelial cells [24], which was performed in [22]. The corresponding reaction system model had 6720 reactions involving 246 entities. Comparing the running time for the context sequence of length 1000 also taken from [22] we obtained that the average time of running brsim was 4.2 s, of HERESY in CPU mode was 10.1 s and the average running time of our Verilog test bench was 7.98 s.

The next performed step was to run the obtained circuit in hardware. We have used a Diligent Basis 3 FPGA board, which features a Xilinx Artix 7 architecture. Since the result of our translation consists only of the circuit simulating each step of the reaction system (the test bench cannot be synthesized in hardware), we had to manually add the circuits allowing to enter the (input) context sequence as well as to save the output result. We used several test reaction systems (including those from Examples 2 and 3) as well as different input/output circuits. For small-size examples (up to 16 inputs and 16 outputs) we used switches and leds present on the board as input and output, allowing to verify the correctness of the simulation. In order to speed-up the computation we also considered input coming from the serial port at 115,200 bits/s. The tests have shown the correctness of the simulation.

Finally, the last tests were performed using an autonomous execution of the system without output and using distributed read-only memory data storage for the input (or context-less models). Under this setup the speed of 100 Mhz (corresponding to the system clock) was achieved. This means that a reaction system model can be simulated at a speed of $10^{8}$ steps per second. Applied to the ErbB model this gives a speed-up of $2.5\times 10^{5}$ with respect to the GPU-based simulator from [22].

Our tests show a low usage of FPGA resources (look-up tables (LUTs) and slices). For example, the ErbB model uses only 186 LUTs and 55 Slices, which corresponds to 0.89% and 0.67%, respectively, of available resources on Basis 3 board. Due to the simple architecture of the system these numbers suggest that a basis 3 board can handle reactions systems having up to 20 K reactants and 500 K reactions. Using a larger board, like VC707 based on Xilinx Virtex-7 architecture, it is possible to increase the size of the simulated system by 15 times.

From the speed point of view, our architecture allows to perform one computation step during one clock tick, so it runs at FPGA main clock speed, which can range from 50 Mhz to 400 Mhz. However, one needs to add the time needed for the input/output procedures as they are usually much slower (except for the input/output which are preloaded in distributed or block RAM). Depending on the size of the context it might be convenient to load it initially into the RAM, or to acquire the input data at each step, e.g., using serial port or peripheral component interconnect express (PCIe) connections.

We would like to remark that the input/output circuits are not system-specific and can be reused for different simulations. However, at the present state they need to be integrated manually in the final hardware design. It would be interesting to complete RStoVerilog compiler with a tool allowing to automate this process.

## Figures and Tables

**Table 1:** The truth table for Example 1.

$q_{1}\left(t\right)$	$q_{2}\left(t\right)$	$x_{1}\left(t\right)$	$q_{1}\left(t+1\right)$	$q_{2}\left(t+1\right)$	$y_{1}\left(t\right)$
0	0	0	1	0	1
0	0	1	1	0	1
0	1	0	0	0	0
0	1	1	0	0	0
1	0	0	1	1	0
1	0	1	0	1	0
1	1	0	0	0	0
1	1	1	0	1	0

**Table 2:** The truth table for Example 4.

$q_{2}\left(t\right)$	$q_{1}\left(t\right)$	x(t)	$q_{2}\left(t+1\right)$	$q_{1}\left(t+1\right)$	y(t)
0	0	0	0	0	0
0	0	1	0	1	0
0	1	0	0	0	0
0	1	1	1	0	0
1	0	0	1	1	0
1	0	1	1	0	0
1	1	0	0	0	0
1	1	1	0	1	1
